# Early Repolarization Augmentation Mimicking Pseudo-Infarction in a Patient With Diabetic Ketoacidosis and Normokalemia

**DOI:** 10.7759/cureus.41546

**Published:** 2023-07-07

**Authors:** Aleksan Khachatryan, Robert D Chow, Hakob Harutyunyan, Vahagn Tamazyan

**Affiliations:** 1 Department of Internal Medicine, University of Maryland Medical Center Midtown Campus, Baltimore, USA; 2 Department of Internal Medicine, Maimonides Medical Center, New York, USA

**Keywords:** brugada syndrome, j point, j wave, pseudo-infarction, diabetic ketoacidosis, early repolarization syndrome, early repolarization pattern

## Abstract

Early repolarization (ER) changes, characterized by J point elevation with or without ST-segment elevation, are dynamic in their presentation and can be exacerbated by factors such as hypothermia, hypercalcemia, vagotonia, and certain medications. There is limited research regarding the mechanism of these changes and the dynamic changes of ER secondary to diabetic ketoacidosis (DKA). This case report highlights the augmentation of early repolarization changes resembling ST-segment elevation myocardial infarction (STEMI) in a patient with DKA that resolved with the treatment of acidosis. The misinterpretation of ER changes on electrocardiogram (ECG) as STEMI or pericarditis may result in the inappropriate utilization of resources, increased patient risk, and elevated morbidity and mortality. Recognition of the potential of DKA to cause ER changes can potentially avoid these unfavorable outcomes.

## Introduction

The early repolarization pattern (ERP) is currently defined as the presence of a J wave in the terminal part of the QRS complex manifested as a notch or slur with J point elevation ≥ 0.1 mV in at least two adjacent leads, with or without ST-segment elevation. Historically considered a benign condition, concern was raised in the early 2000s regarding the potential of ERP to be a harbinger of future arrhythmias [1]. Later, in 2008, compelling evidence emerged correlating ERP with ventricular tachycardia (VT)/ventricular fibrillation (VF), both of which can precipitate sudden cardiac death (SCD) [2,3]. If ERP is found in a patient who either survived VT/VF or experienced SCD with no identifiable alternative cause of cardiac arrest, it is termed early repolarization syndrome (ERS) [4].

DKA is a life-threatening complication of predominantly type 1 diabetes mellitus (DM) and has been shown to be associated with various ECG changes [5]. The most prevalent among the reported ECG abnormalities are pseudo-infarction [6-8] and Brugada patterns [9,10]. These ECG changes are primarily attributed to metabolic disturbances, with hyperkalemia and acidosis frequently coexisting in the majority of reported cases [10]. The ECG manifestations of Brugada patterns as well as that of Brugada syndrome (BrS) [11] in association with DKA have been extensively documented. Although BrS and ERP are described as parts of a continuum termed J-wave syndromes, the current literature sheds limited insight regarding the relationship between the dynamic changes in ERP and the presence of acidosis, particularly in the context of DKA-induced acidosis. Our case highlights the clinical importance and diagnostic challenges for clinicians when a pseudo-infarction pattern is present on ECG in patients with DKA and normokalemia, particularly if exaggerated ERP mimicking STEMI exists.

## Case presentation

A 23-year-old man with a documented history of ERP, uncontrolled type 1 DM, and mild intermittent asthma presented with diffuse abdominal cramps, dizziness, nausea, vomiting, polyuria, polydipsia, and generalized weakness. The patient also reported dyspnea on exertion and elevated systolic blood pressure readings to 200s over the course of the prior week. Notably, the patient denied chest pain, paroxysmal nocturnal dyspnea, orthopnea, lower extremity swelling, syncope, palpitations, fever, cough, recent infections, or joint pain. The patient had a similar presentation a month ago and was diagnosed with DKA secondary to type 1 DM. He was discharged on a regimen of glargine insulin 25 units nightly and insulin aspart 12 units before meals but was not adherent due to a lack of syringes. The family history was notable for DM in both parents and SCD in his mother at the age of 47. The patient denied smoking, illicit drug use, or alcohol consumption.

Upon examination, the patient's vital signs were as follows: temperature of 36.9°C, heart rate of 123 beats per minute, blood pressure of 125/84 mmHg, respiratory rate of 16 breaths per minute, and oxygen saturation of 98% on room air. The patient did not appear to be in distress. Mucosal membranes were dry. The cardiovascular examination was unremarkable, with the absence of JVD, friction rubs, murmurs, or gallops. There was diffuse abdominal tenderness without rebound or guarding. Mental status was preserved.

The initial laboratory findings revealed significant abnormalities, including a blood glucose level of 605 mg/dL (normal 74-106 mg/dL), venous pH of 7.039 (normal 7.320-7.420), serum anion gap of 29 mmol/L (normal 4-16 mmol/L), and serum bicarbonate of 7 mmol/L (normal 21 - 30 mmol/L). Additionally, the urine analysis indicated the presence of ketones and glucosuria. The remaining laboratory results demonstrated serum potassium of 4.8 mmol/L (normal 3.5-5.1 mmol/L), serum sodium of 134 mmol/L (normal 137-145 mmol/L), serum magnesium of 1.9 mg/dL (normal 1.6-2.3 mg/dL), serum calcium of 9.8 mg/dL (normal 8.4-10.2 mg/dL), and serum creatinine of 0.92 mg/dL (normal 0.70-1.50 mg/dL).

The initial ECG demonstrated accelerated junctional rhythm with a heart rate of 74 beats per minute. Notably, J waves were observed in leads I, aVL, V5, and V6, with J point elevation of 2 mV in lead I, 1 mV in lead aVL, and 3.5 mV in leads V5 and V6. There was an upward-sloping ST-segment elevation of 2 mm in leads II, III, aVF, V5, and V6. Troponin levels were at 0.039 ng/mL (reference range ≤0.034 ng/mL) potentially secondary to lab variability. A chest X-ray did not reveal any significant abnormalities, and transthoracic echocardiography showed no signs of regional wall motion abnormalities or pericardial effusion.

**Figure 1:**
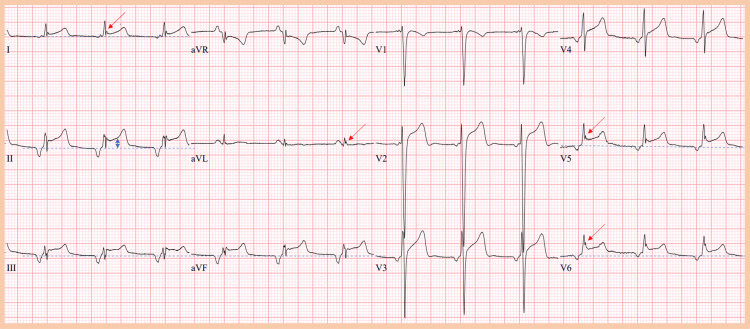
Initial ECG The initial ECG showed an accelerated junctional rhythm with a heart rate of 74 beats per minute. Notably, J waves were observed in leads I, aVL, V5, and V6, as indicated by red arrows. To assess Jp amplitude and ST-segment elevation, dotted blue lines were employed as imaginary isoelectric lines in leads I, II, III, aVF, V5, and V6. Jp amplitude was 2 mV in lead I, 1 mV in lead aVL, and 3.5 mV in leads V5 and V6. There was an upsloping ST-segment elevation of 2 mm in leads II, III, aVF, V5, and V6. The ST-segment elevation in lead II is depicted by the blue double arrow.

**Figure 2:**
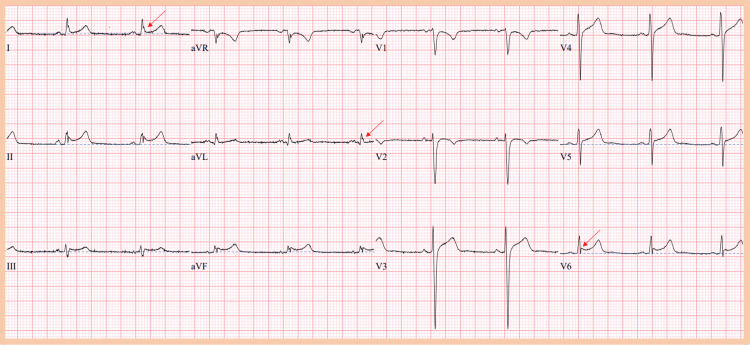
Second ECG The second ECG demonstrates spontaneous conversion to sinus rhythm with a heart rate of 61 and corresponding pH of 7.274. There are now J waves in I, aVL, and V6 as pointed out by the red arrows. ST-segment elevation has significantly improved in leads II, III, aVF, V5, and V6 and now is 1 mm in the II, aVF, V5, and V6 leads. J wave is not present in the V5 lead and the Jp amplitude has significantly improved in lead V6. The dotted blue lines are depicted for the evaluation of ST-segment elevation and Jp amplitude.

**Figure 3:**
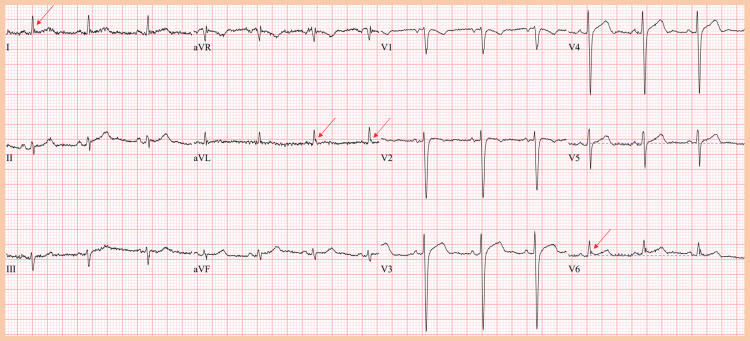
Final ECG The final ECG demonstrates complete resolution of ST-segment elevation in all the leads with normalization of the venous pH to 7.321. The J waves are notable in leads I, aVL, and V6 with a Jp amplitude of around 1 mV.

A diagnosis of DKA was made and the patient was promptly started on intravenous fluids and insulin infusion. Serial laboratory testing and ECGs were performed as part of routine monitoring. Figures 1-3 are the initial and final ECGs during the patient’s hospitalization. With the resolution of DKA and normalization of serum pH, Figure 3 depicts the resolution of the ST-segment elevation and improvement of the J point amplitude.

## Discussion

ERP was first described in 1936 by Shipley and Hallaran [12]. The discovery of the J wave dates back to 1938 when Tomaszewski observed it in an accidentally frozen human. Later, in 1953, the J wave was described by Osborn as a “current of injury” on hypothermic canine models, a phenomenon commonly referred to as the "Osborn Wave." It was unclear at the time whether the cause of the J wave was hypothermia or acidosis [13]. However, Osborn later acknowledged the inaccuracy of the measurement of pH in his experiments [14]. Subsequently, Santos and Kittle used an animal model to clarify the underlying cause of the J wave, concluding that its appearance was induced by hypothermia rather than acidosis [15].

In patients presenting with ERP, the differential diagnoses include STEMI, pericarditis, and BrS. Given our patient's age and absence of risk factors, the baseline risk of coronary artery disease was low. Furthermore, the patient did not exhibit chest pain, and the echocardiography results did not reveal regional wall motion abnormalities. Troponin levels were unremarkable, and the ECG changes improved rapidly with the treatment of acidosis. These findings collectively made acute coronary syndrome less likely. Pericarditis was also unlikely, given the absence of typical chest pain, characteristic friction rub upon auscultation, and normal echocardiography results. Additionally, the absence of prominent J waves in leads V1-V3 rules out BrS as the cause of the observed J point/ST-elevation in this patient. Taking into account all of these findings and the absence of electrolyte abnormalities, the most probable underlying cause of the accentuated ERP, manifested as J point/ST elevation, can be attributed to acidosis secondary to DKA.

**Figure 4:**
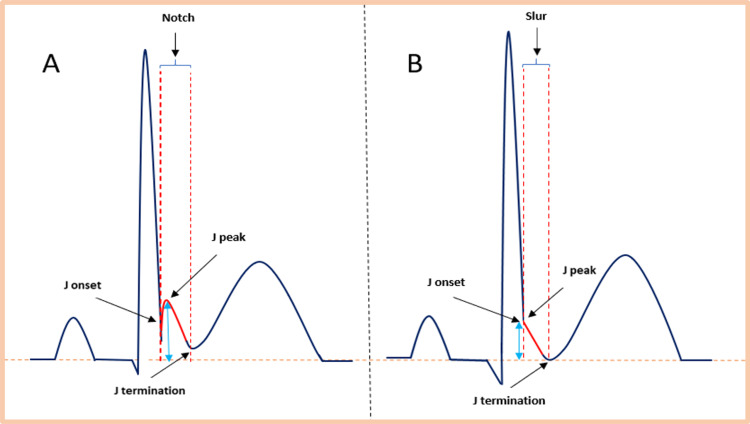
ERP on the surface ECG The red solid color illustrates the J wave on the downslope of a prominent R-wave as either a notch (A) or a slur (B). J onset and J peak are the same points for the slur as depicted in Figure 4B. The light blue double arrow indicates the amplitude of the J point. Current criteria for a diagnosis of ER include: 1. End-QRS notch or slur on the downslope of a prominent R-wave and 2. J point ≥0.1 mV in two or more contiguous leads, excluding leads V1-V3, and 3. QRS duration <120 ms. ERP: early repolarization pattern

Given the historical perception that ERPs are relatively benign phenomena, various definitions have been proposed, resulting in inconsistent data and confusion during data interpretation. As awareness grew regarding the association between ERP and VT/VF, the need for a standardized definition became imperative. Macfarlane et al. (2015) introduced a unified definition of ERP, attempting to facilitate a common understanding and future research in this area [16]. They provided a comprehensive elucidation of J wave variations, depicted as end-terminal QRS notch or slur. To accurately quantify the J point amplitude in the case of a notch, it was suggested that it should be measured from the J peak (Jp) to the isoelectric line. Similarly, for a slur, the J wave amplitude should be measured from the beginning of a slur or J onset (Jo) point to the isoelectric line. It is important to note that the terms J onset and J peak are electrocardiographically the same point when evaluating the J wave amplitude, in case of a slur. Please refer to Figure 4 for a visual representation of these measurements.

Patton et al. suggested that the general term ER encompasses ST-segment elevation in the absence of chest pain, terminal QRS slurring, or terminal QRS notching; however, researchers who use the term ER should clearly specify to which of the aforementioned ECG patterns they are specifically referring [17].

There have been several similarities between BrS and ERP, as they share the same pathophysiologic platform, risk factors, and outcomes in terms of malignant arrhythmias. It has been shown that quinidine, cilostazol, and isoproterenol reverse the repolarization abnormalities that are observed in both BrS and ERP. BrS and ERP are often grouped together under the umbrella term J wave syndrome [18,19]. One anatomic difference is, however, that BrS involves the right ventricular outflow tract and ERP includes the left ventricular lateral, inferior, or inferolateral walls.

The prevalence of ERP ranges from 5-18%. ERP is more frequently encountered in males, African Americans, and young individuals. A large study conducted in Finland found that the prevalence of inferolateral ERP with J point elevation ≥ 1 mm in two adjacent leads is 5.8% [20].

**Figure 5:**
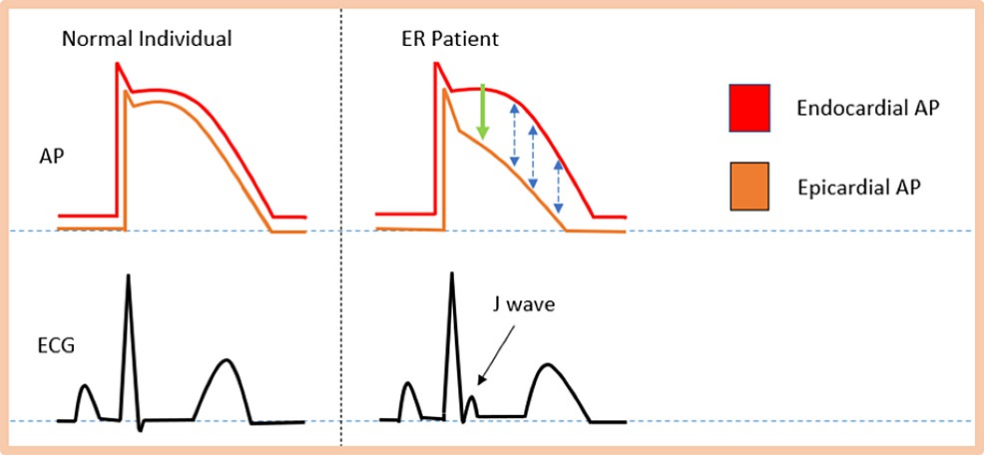
Illustration of the possible mechanisms underlying J-wave occurrence Action potential from epicardium and endocardium from normal individuals and ER patients, as well as the respective ECGs, are shown. A prominent phase 1 notch and the loss of the epicardial dome in phase 2 (green arrow) results in the generation of a transmural gradient (dashed double arrows) and the appearance of the J wave on the surface ECG. AP: action potential; ECG: electrocardiogram; ER: early repolarization

Numerous underlying mechanisms have been proposed to explain the occurrence of the J wave in ERP. Among these theories, the most widely accepted one is related to the interplay between inward sodium (Na+), calcium (Ca2+), and transient outward (Ito) potassium (K+) channels, resulting in an accelerated net outward current [19,21]. The heterogeneous distribution [22] and higher density of Ito channels in the epicardium generates amplified net outward current during phase 1 of repolarization, which results in the development of a transmural voltage gradient between the epicardial and endocardial layers. This voltage gradient manifests as a J wave, which appears as a notch or slur on an ECG, as depicted in Figure 5. If the voltage gradient arises in the later phases of the repolarization curve, it then gives rise to ST-segment elevation [21]. Conditions such as hypercalcemia, hypothermia, vagotonia, certain medications, and probably acidosis that either negatively affect inward current through Na+ or Ca2+ channels or increase outward Ito current may accentuate the expression of the J wave. Furthermore, the pathogenesis of ERP has been associated with a number of genetic mutations in genes such as *KCNJ8, CACNA1C, CACNB2, CACNA2D1*, and *SCN5A*. Collectively, these mutations enhance net outward current, contributing to accelerated repolarization in the epicardial layers [23-27]. The augmentation of the J wave has been associated with increased risks of VT/VF in several studies [28,29]. Therefore, it is crucial for clinicians to be aware of the heightened potential for malignant arrhythmias in ERP patients who develop acidosis.

In general, patients with ERP are typically asymptomatic. However, patients with ERS are either survivors of VT/VF or have a history of syncope attributed to ERS.

The diagnosis of ERP depends on specific ECG changes, according to established definitions. It is important to note that ST-segment elevation is not considered mandatory for the diagnosis of ERP. Antzelevitch et al. described three types of ERP that highlight the anatomic localization and the associated risk of arrhythmia [30]. Type 1 ERP, which is characterized by its presence in lateral ECG leads, exhibits the lowest risk of arrhythmia. Type 2 ERP refers to the involvement of inferior or inferolateral leads while type 3 ERP is defined by the presence of ERP in all leads, including lateral, inferior, and right precordial leads. Notably, type 3 ERP carries the highest risk of arrhythmia.

The treatment of asymptomatic ERP patients is not well-validated. Currently, there are limited data supporting the use of implantable cardioverter-defibrillators (ICDs) (Class IIb) in patients with high-risk features such as a prominent J wave or a family history of SCD. The ICD implantation is recommended for all ERS survivors or documented VT/VF, regardless of the presence or absence of syncope. In the acute management of VT/VF, isoproterenol and quinidine have been shown to be effective. For patients who have an ICD implanted but are experiencing frequent shocks, suppressive therapy with isoproterenol or quinidine is recommended.

While rare case reports exist regarding DKA uncovering BrS [11], limited data are available regarding ECG changes in DKA patients with pre-existing ERP. In the majority of reported cases, DKA-associated ECG changes are described when hyperkalemia and acidosis coexist [10]. However, in our patient, pure acidosis was observed without hyperkalemia, which is a rarely described phenomenon in the literature.

## Conclusions

In conclusion, it is important to recognize that DKA may accentuate early repolarization changes in patients with pre-existing ERP. This can manifest as the elevation of the J point and ST-segment simulating the characteristic features of a pseudo-infarction pattern on ECG. Awareness of this phenomenon can assist in managing diagnostic challenges and prevent unnecessary escalation of care.
